# Targeting Apoptosis Pathways in Cancer with Alantolactone and Isoalantolactone

**DOI:** 10.1155/2013/248532

**Published:** 2013-10-27

**Authors:** Azhar Rasul, Muhammad Khan, Muhammad Ali, Jiang Li, Xiaomeng Li

**Affiliations:** ^1^The Key Laboratory of Molecular Epigenetics of MOE, Institute of Genetics and Cytology, Northeast Normal University, Changchun 130024, China; ^2^Dental Hospital, Jilin University, Changchun 130041, China; ^3^Department of Zoology, University of the Punjab, Lahore 54590, Pakistan; ^4^Institute of Molecular and Biotechnology, Bahauddin Zakariya University, Multan 60800, Pakistan

## Abstract

Alantolactone and isoalantolactone, main bioactive compounds that are present in many medicinal plants such as *Inula helenium, L. Inula japonica, Aucklandia lappa, Inula racemosa,* and *Radix inulae*, have been found to have various pharmacological actions including anti-inflammatory, antimicrobial, and anticancer properties, with no significant toxicity. Recently, the anticancer activity of alantolactone and isoalantolactone has been extensively investigated. Here, our aim is to review their natural sources and their anticancer activity with specific emphasis on mechanism of actions, by which these compounds act on apoptosis pathways. Based on the literature and also on our previous results, alantolactone and isoalantolactone induce apoptosis by targeting multiple cellular signaling pathways that are frequently deregulated in cancers and suggest that their simultaneous targeting by these compounds could result in efficacious and selective killing of cancer cells. This review suggests that alantolactone and isoalantolactone are potential promising anticancer candidates, but additional studies and clinical trials are required to determine their specific intracellular sites of actions and derivative targets in order to fully understand the mechanisms of therapeutic effects to further validate in cancer chemotherapy.

## 1. Introduction

Plants have a long history of use in the treatment of cancer and there are more than 3000 plant species that have been used in the treatment of cancer [1]. There is a continued interest in the investigation of extracts of microorganisms, terrestrial plants, and marine life forms to search for anticancer compounds [2]. Herbal medicines, as an important novel source with a wide range of pharmaceutical potential, are being used for the treatment of human ailments including almost all kinds of cancer [3]. Indeed, molecules derived from natural sources have played and continued to impart a dominant role in the discovery of compounds for the development of conventional drugs for the treatment of most human diseases [4].

Many herbal medicines, such as paclitaxel, camptothecin, vinca alkaloids, and etoposide hold great potential as promising agents for the treatment of cancer [2]. As these and many other natural products have traditionally provided a rich source of drugs for cancer, so plants have always been considered as important sources of natural products [5]. Although different approaches are available for the discovery of novel and potential therapeutic agents, even then natural products from medicinal plants are still one of the best reservoirs for novel agents with new medicinal activities [6]. Many medicinal plants belong to genera of family Asteraceae (Compositae), and angiosperms are reported for sesquiterpene lactones (SLs) [7]. SLs belong to a representative class of the biologically active components [8–10]. These are known to possess cytotoxic, anti-inflammatory, antiviral, antifungal, antineoplastic, and antiparasitic activities [11–13]. 

Sesquiterpene isolates (alantolactone, isoalantolactone, and 5-epoxyalantolactone present in *I. helenium*) present a significant increase of QR activity in liver, kidney, small intestine, and stomach [14] and showed antiproliferative activities against MK-1, HeLa, and B16F10 cells [15]. The extensive structural diversity and potential biological activities of this class have ignited further interest among the chemists and biologists. This review summarizes the recent researches on alantolactone and isoalantolactone focusing on anticancer activity. The literature was screened through various e-sites including PubMed, Scopus, and Elsevier Science Direct Journal. Access to the Elsevier Science Direct Journal was made possible through library of Northeast Normal University, Changchun, China. The literature search mainly focused on recent advances and additional manual searches were carried out on relevant medical journals and the Google Search Engine. Key words used for search were “alantolactone,” “isoalantolactone,” “anticancer activity,” “cancer therapy,” “cytotoxicity,” and “medicinal plants.” The data collected is from primary sources and/or from data that superseded earlier work.

## 2. Natural Sources

**Figure 1:**
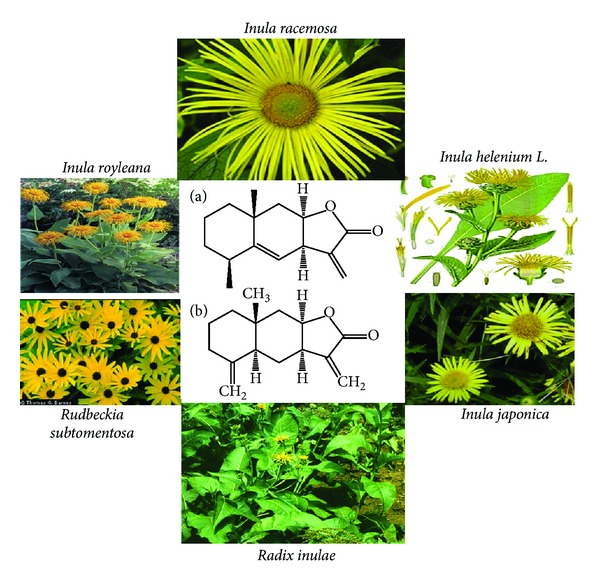
Chemical structure of alantolactone (a) and isoalantolactone (b) and natural sources.

Alantolactone and isoalantolactone (Figure 1) have been isolated from many species of plants, including *Inula Helenium L. *[14, 16–22], *Inula japonica *[23], *Aucklandia lappa*, [24], *Inula racemosa* Hook. F Pushkarmula (Compositae) [18], *Inula royleana* [21], *Rudbeckia subtomentosa Pursh* [25], *Radix inulae,* and from *Liuwei Anxian San* [26]. The phytoconstituent from these plants was extracted by HPLC, micellar electrokinetic chromatography (MEKC), microemulsion electrokinetic chromatography (MEEKC), and their structures were elucidated on the basis of NMR analysis.

## 3. Biological Activities

Despite the limited data that is available for their biological applications, alantolactone and isoalantolactone have sound medicinal importance. Studies on alantolactone and isoalantolactone showed larvicidal activities [25, 27]. It presents its capability as a better sensitizer for allergic contact dermatitis [20]. Alantolactone and isoalantolactone have been reported for their wide spectrum of biological effects, including antifungal, anthelmintic activities, [28, 29], antimicrobial activities [22, 30], anti-inflammatory activities [24, 31], antitrypanosomal activities [31], and antiproliferative effects on several cancer cell lines, such as colon, melanoma, ovary, prostate, lung, and leukemia [15, 32]. In addition, isoalantolactone protects against *Staphylococcus aureus* pneumonia [33]. This review will focus on the mechanisms by which alantolactone and isoalantolactone act on apoptosis pathways in cancer that have been characterized thus far, including other pathways like caspase-mediated common pathway, and regulation of apoptosis-related proteins. These breakthrough findings may have important implications for targeted cancer therapy and modern applications of alantolactone and isoalantolactone.

## 4. Targeting Apoptosis Pathways in Cancer with Alantolactone and Isoalantolactone

Apoptosis is defined as an extremely synchronized mode of cell death. It is characterized by distinct morphological features, including chromatin condensation and nuclear fragmentation [34, 35]. The importance of signaling has been recognized in cell regulation during normal and disease [36, 37]. Accumulated data [38–45] suggest that various anticancer chemopreventive agents can induce apoptosis which in turn induce death in cancerous cells. Fulda and Debatin, 2006 [46] reported that two main pathways are involved to initiate apoptosis, the intrinsic or mitochondrial, and the extrinsic or death receptor pathway; both pathways eventually activate the same effectors caspases and apoptosis effectors molecules. Fas, TRAIL, and TNF receptors are highly specific physiological mediators of the extrinsic signaling pathway of apoptosis. Cross-linking of death receptors either with their natural ligands (e.g., FasL, TRAIL, and TNF-*α*) or with agonistic antibodies (such as anti-APO-1) induces a sequential activation of availability of high throughput assays, based on the above-said molecular targets which has enhanced the process of drug discovery for regulating these pathways [47]. Extensive studies have revealed that alantolactone and isoalantolactone both induce apoptosis in various tumor cells. Alantolactone isolated from *Inula helenium* (Compositae), a traditional Chinese medicinal herb, provides an effective inhibitory activity for cell growth against MK-1, HeLa, B16F10, and K562 cell lines [15, 48]. Many other human cancer cell lines, including U87 glioma cells [49], Bel-742, SMMC-7721 and HepG2 liver cancer cells [50, 51], PANC-1 pancreatic carcinoma cells [52], A59 lung cancer cells [53], colon adenocarcinoma HCT-8 cells [45], CNS cancer cell line SF-295, leukemia HL-60 [32], Hepa1c1c7 cells, BPRc1 Hepatic cancer cells [20], and HCT-8 colon cancer cells [45], have also been reported for apoptosis caused by alantolactone. Moreover, it has also been reported that isoalantolactone induces anti-inflammatory activity against lipopolysaccharide (LPS)-stimulated RAW 264.7 cells [24]. 

Isoalantolactone, isolated from *Inula helenium*, exhibits antiproliferative activities against various cancer cells such as MK-1, HeLa, and B16F10 cells [15]. The increase in the activity of QR biomarker enzyme of phase 2 antioxidant enzymes is attributed to the presence of the isoalantolactone. This change in the activity of an enzyme reciprocally affects the expression of other detoxifying/antioxidant enzymes and thus enhanced the levels of GR, GGCS, GST-Pi, and HO-1 [14, 19, 20]. Contrary to its cytotoxic reports, *in vitro* studies in CD1 mice at IAL dose of 100 mg/kg body weight showed that IAL does not induce any acute or chronic toxicity in liver and kidney thus suggesting that isoalantolactone may be a safe chemotherapeutic candidate for the treatment of human pancreatic carcinoma [52].

This review further focuses on the mechanisms by which alantolactone and isoalantolactone act on apoptosis pathways in the different types of the cancer cells that have been characterized. The different signaling pathways (death receptor-mediated pathway, mitochondria-mediated pathway, caspase-mediated common pathway, and regulation of apoptosis-related proteins) that are highly related to the presence or absence of alantolactone and isoalantolactone have been discussed briefly.

### 4.1. Targeting Cancer Cells by Mitochondria-Mediated Apoptosis

Mitochondria have become an important component of the apoptosis execution machinery, which contain proapoptotic proteins (e.g., cytochrome c) [34]. It has been elucidated that the depolarization of the mitochondrial membrane potential results in the mitochondrial swelling and subsequent release of cytochrome c from the intermitochondrial membrane space into the cytosol [54]. It is becoming increasingly apparent that the mitochondria play a fundamental role in the processes leading to cell death [55]. Several reports reveal that the effects of alantolactone on the intrinsic pathway of apoptosis have been examined in many cell lines, including human leukemia K562 cells [56, 57], human hepatoma HepG2 cells [50, 51], and glioblastoma U87 cells [49]. Isoalantolactone significantly reduced the mitochondrial potential (ΔΨm) in pancreatic carcinoma PANC-1 cells [52]. We recently showed that treatment with alantolactone and isoalantolactone induced apoptosis by increasing translocation of cytochrome c from mitochondria to cytosol and activation of caspase-3 in HepG2, PANC-1, and U87 cell lines [49, 50, 52]. In addition, it has been investigated by many researchers that alantolactone initiated apoptosis through cytochrome c/caspase-3/PARP in leukemia K562 cells [56, 57]. 

### 4.2. Targeting Cancer Cells by ROS-Mediated Apoptosis

ROS are well known mediators of intracellular signaling of cascades. The excessive generation of ROS can induce oxidative stress, loss of cell functioning, and apoptosis [58]. The interaction between members of the Bcl-2 protein family regulates the apoptosis through mitochondrial pathway. ROS can also be involved in the process of lipid peroxidation and/or the cross-linking of thiol groups in proteins; both of these processes can induce the opening of the mitochondrial permeability transition pore (PTP) [59, 60]. Current studies demonstrated that ROS plays an important role in depolarizing mitochondria and alantolactone- and isoalantolactone-induced apoptosis in different types of cancer cells [49, 50, 52]. Moreover, the elevation in the activities of caspases suggests that apoptosis of cancer cells induced by n-hexane fraction of sesquiterpene is mediated through activation of proteases [32]. These proteases act on specific substrates leading to the degradation of PARP and other cytoskeletal proteins, which are responsible for many of the morphological and biochemical features of apoptosis in cancer cells [32, 49–52]. Activated caspases might target the permeabilized mitochondria, resulting in the loss of mitochondrial membrane potential concomitant with increased production of ROS, and this activity eventually causes disruption of membrane integrity [58]. In addition, our findings also demonstrated the sensitivity of tumor cells to alantolactone that appears as a result of GSH depletion and ROS production [49]. Further studies reveal that apoptosis induction more or less depends on many factors like increase in ROS, oxidation of cardiolipin, reduced mitochondrial membrane potential, and release of cytochrome c [49]. Khan et al. [52] have explained the involvement of ROS in isoalantolactone-mediated apoptosis. The specific ROS inhibitor, N-Acetyl Cysteine (NAC) restored cell viability and completely blocked apoptosis mediated by isoalantolactone in PANC-1 cells. The activation of p38 MAPK and Bax is directly dependent on ROS generation.

### 4.3. Targeting Cancer Cells by Caspase-Mediated Apoptosis

The caspases, a family of cysteine proteases, are one of the focal executors of the apoptotic process via triggering of the death receptors and mitochondrial pathways to accomplish the programmed cell death [61].

 Caspases are present in the form of inactive zymogens that are activated during apoptosis. Among them, caspase-3 is a frequently activated death protease, catalyzing the specific cleavage of many key cellular proteins [62, 63]. Several studies reveal effects of alantolactone and isoalantolactone on expression of caspases. We and others report that alantolactone induces apoptosis in HepG2 cells by activation of caspase-3 and modulating the level of Bcl-2 protein family [50, 51]. Moreover, Lei et al. demonstrate that caspase-8 activation is associated with alteration of Bid in liver cancer cells [51]. Furthermore, the cleavage of specific substrates for caspase-3, poly (ADP-ribose) polymerase (PARP), is involved in alantolactone-induced apoptosis in various cancer cells [49–51, 56, 57]. Interestingly, during early apoptosis the release of caspase activating proteins is primarily regulated by Bcl-2 protein family, an important regulator of apoptosis. Among these proteins, overexpression of proapoptotic protein Bax induces the release of cytochrome c from the mitochondria. It is considered an important event that affects apoptosis mediated by mitochondrial pathway [64].

### 4.4. Targeting Cancer Cells by Regulating Apoptosis Related Proteins

**Figure 2:**
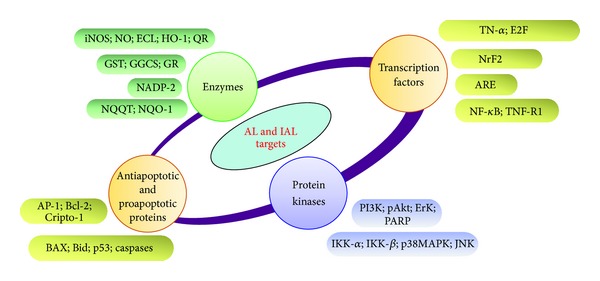
Alantolactone (AL) and isoalantolactone (IAL) target multiple cell signaling pathways. AL and IAL inhibit various targets including transcription factors, enzymes, growth factors and its receptors, kinases, and antiapoptotic proteins. Note: iNOS, inducible nitric oxide synthase; NO, nitric oxide; HO-1, heme oxygenase-1; GST, glutathione-S-transferase; NADP-2; NADH quinone oxidoreductase 1 NAD(P)H dehydrogenase, quinone1; NF-*κ*B, nuclear factor kappa B; TNF-R1, TNF receptor-associated factor-1; AP-1, activating protein-1; Bcl-2, B-cell lymphoma protein 2; BAX, Bcl-2-associated X protein; Bid, BH3-interacting domain death; GST, glutathione-stransferase; GR, glutathione reductase; GGCS, gamma-glutamyl cystein synthetase; PI3 K, phosphoinositide 3-kinase; ERK, extracellular receptor kinase; PARP, poly (ADP-ribose) polymerase; IKK, I*κ*B-*α*, kinase-alpha; IKK, I*κ*B-*β*, kinase-beta; p38MAPK, mitogen-activated protein kinases; and JNK, c-Jun N-terminal kinases.

There are well-known targets at the signaling levels that have been identified to proliferate cancer cells. It is believed that in normal cells certain cellular signals control and regulate their growth and all other growth mechanisms. When these signals are altered due to various mutations that prevent cells from undergoirs apoptosis, normal cells are transformed into cancerous cells and undergo hyperproliferation. Therefore, to arrest cancerous cell proliferation, regulation of apoptosis and its signaling pathways play a critical role [65–67]. Next, we reviewed the effects of various signaling pathways that have been reported in alantolactone- and isoalantolactone-induced apoptosis have been summarized in Figure 2 and Table 1.

#### 4.4.1. p53

p53, a transcription factor, is considered as a “guardian of the genome” plays a crucial role in the regulation of cell cycle progression, checkpoint activation [68], and assume that this may favor apoptosis [69] and repair of DNA damage [70]. As a multitasking agent it is important for the suppression of tumor formation, as well as for mediating the cellular responses to many standard DNA damage inducing cancer therapies [71, 72]. It has also been documented that targeting p53 pathway, which is frequently deregulated in cancer, by anticancer agents could result in efficacious and selective killing of cancer cells [39]. We and others have shown that alantolactone significantly increased the expression of p53 in HepG2 cells [50, 51] with concomitant increase of its downstream target, the cyclin-dependent kinase inhibitor p21 in adriamycin (ADR)-resistant human erythroleukemia cell line K562/ADR [57]. This behavior may lead to cell cycle arrest and up regulate Bax expression [50, 51, 57]. In addition, it also documented that alantolactone induced cell-cycle arrest in the G2/M phase via down-regulation of cyclin B1 and cyclin-dependent protein kinase 1 [57]. Interestingly, our unpublished data showed that isoalantolactone isomer of alantolactone can induce apoptosis in prostate cancer PC3, null p53 cells, which indicate that isoalantolactone may induce p53-independent apoptosis. Further studies are required to define an obvious role of p53 in alantolactone- and isoalantolactone-induced apoptosis and cell cycle arrest in cancer cells.

#### 4.4.2. NF-*κ*B

NF-*κ*B1 (p50), NF-*κ*B2 (p52), c-Rel, RelB, and RelA (p65) collectively constitute NF-*κ*B family [73]. NF-*κ*B and other signaling pathways that are involved in its activation are highly significant in tumor development [74]. This assumption relies on inferred evidences, such as the frequent presence of constitutively activated NF-*κ*B in diverse solid malignancies and the well established ability of NF-*κ*B to upregulate production of key proinflammatory cytokines and enzymes. Other agents such as TNF-*α*, IL-1, IL-6, and COX-2,5 in an inflammatory microenvironment are involved in tumor progression, incursion of adjoining tissues, angiogenesis, and metastasis [75]. The expression of many genes is also regulated by NF-*κ*B. This regulation ultimately suppresses tumor cell death, enhances epithelial to mesenchymal transition, and also stimulates tumor cell cycle progression [75]. NF-*κ*B inhibits apoptosis by inducing the expression of Bcl-2 family members and caspases inhibitor [76]. NF-*κ*B helps to induce proteolytic matrix metalloproteinases (MMPs) enzyme that promote tumor invasion. Moreover, IKKa promotes metastasis in prostate cancer through inhibition of mammary serine protease inhibitor (maspin) [77, 78]. NF-*κ*B activation also stimulates angiogenesis, probably by inducing expression of IL-8 and vascular endothelial growth factor (VEGF) [74]. In short, these studies validated NF-*κ*B as a unique and novel target for cancer therapy. These studies also stimulate the motivation and dedicated insight for developing small-molecule NF-*κ*B inhibitors. It is consider as a transcription factor activated in various neoplasms [79]. Detailed study of the literature validated that NF-*κ*B signaling pathways played critical role in a wide variety of physiological and pathological processes. It is involved in promoting cell survival through induction of target genes. The products of cell survival inhibit components of the apoptotic machinery in normal and cancerous cells [80]. NF-*κ*B protein stimulates the cell survival and promotes cell proliferation, and its increased activity is positively associated with many cancer types [81, 82]. Hence, inhibition of NF-*κ*B can induce apoptosis in cancer cells, offering a promising strategy for the treatment of different malignancies [39].

Many studies have been carried out whether alantolactone treatment inhibits expression of NF-*κ*B or not. Alantolactone is reported to affect nuclear factor *κ*B (NF-*κ*B) signaling, the phosphorylation of inhibitory *κ*B (I*κ*B)-*α*, and I*κ*B kinase (IKK) is inhibited by alantolactone, which subsequently translocates the p65 and p50 NF-*κ*B subunits to the nucleus [49]. Alantolactone exerts an anti-inflammatory effect in LPS-stimulated RAW 264.7 cells by suppressing NF-*κ*B activation and MAPKs phosphorylation via downregulation of the MyD88 signaling pathway as well [24]. Another study demonstrates that alantolactone treatment on human liver cancer cells decreases NF-*κ*B/p65 levels in a dose-dependent manner [51]. Using immunoblot analysis, Chun et al. [24] also observed that alantolactone treatment of cells resulted in a significant decrease in NF-*κ*B/p65 protein. Other previous reports suggested that NF-*κ*B is also an important transcription factor in regulating the expression of iNOS, COX-2, and inflammatory cytokines [75]. It was observed that alantolactone suppresses the expression of iNOS and COX-2 through the attenuation of the DNA-binding activity of the transcription factor NF-*κ*B [24]. However, the effects of alantolactone on NF-*κ*B target genes, including IAP1, IAP2, XIAP, cFLIP, survivin, and TRAF1, have not been elucidated yet, while isoalantolactone is not reported yet to affect nuclear factor *κ*B (NF-*κ*B) signaling.

#### 4.4.3. PI3K-Akt

Phosphatidylinositol 3-kinase/Akt pathway is reported to regulate a number of cellular processes, such as cell proliferation, cell growth [83], apoptosis, and rearrangement of cytoskeleton [84]. As an important intracellular pathway it is frequently overexpressed in a wide variety of epithelial malignancies [85]. It is supposed that the overexpression of Akt is associated with poor prognosis, tumor progression, and resistance to systematic therapy. In gastric cancer cells phosphorylation of Akt mainly upregulates the expression of Bcl-2 and downregulates the expression of Bax [85]. As phosphatidylinositol 3-kinase/Akt pathway is considered a main target of most anticancer agents, recently many researchers have focused on the PI3K/Akt pathway as a potential target for therapeutic strategies against cancer. Studies have shown that alantolactone attenuated the phosphorylation of Akt and inhibited the expression of MyD88 and TIRAP proteins associated with the MyD88-dependent pathway [24]. The effect of alantolactone on the phosphatidylinositol 3-kinase (PI3K)-Akt pathway is associated with the MyD88-dependent pathway [24]. These findings showed that AL rapidly induces the phosphorylation of Akt after the stimulation and it can be used as a potent inhibitor against cancer cells.

#### 4.4.4. Nuclear Factor-E2-Related Factor 2 (Nrf2)

Nuclear factor-E2-related factor 2 (Nrf2) is a potential molecular target for cancer chemoprevention by natural compounds [86]. It has been documented that alantolactone stimulated the nuclear accumulation of Nrf2 in the presence of inhibitor phosphatidylinositol 3-kinase (PI3K). Nuclear translocation of Nrf2 is also activated by isoalantolactone. With increasing the dose of isoalantolactone the nuclear level of Nrf2 was enhanced. In addition, isoalantolactone is reported as a potential candidate for chemoprevention, and by stimulating the accumulation of NrF2 in the nucleus it acts as potent phase 2 enzyme inducer. After translocation in nucleus, Nrf2 by interacting with ARE sequences plays a major role in transcriptional activation of phase 2 detoxification enzymes. Like most phase 2 enzyme inducers, isoalantolactone also stimulates nuclear translocation of Nrf2 in Hepa1c1c7 and BPRc1 cells. Isoalantolactone also found to induce QR in a dose-dependent manner and stimulates translocation of Nrf2 in the cells [19, 20].

#### 4.4.5. Cripto-1 and Activin Signaling Pathway

The deregulation of activin signaling contributes to tumor formation. Activin signaling is blocked in cancer cells due to the complex formed by Cripto-1, activin, and activin receptor type II (ActRII). The alantolactone treated mammalian hybrid system suggested that it induced activin/SMAD3 signaling in human colon adenocarcinoma HCT-8 cells. Furthermore, the antiproliferative function of alantolactone is activin/SMAD3 dependent, thus alantolactone performs its antitumor effect by interrupting the interaction between Cripto-1 and the activin receptor type IIA in the activin signaling pathway [45].

#### 4.4.6. STAT3

Signal transducer and activator of transcription 3 (STAT3) has been implicated in many processes including development, differentiation, immune function, proliferation, survival, and epithelial to mesenchymal transition (EMT) [87, 88]. In addition, constitutive activation of STAT3 has been reported in many cancers [87–93]. During EMT many proteases are upregulated and cell adhesion molecule expression is altered to allow for more invasive phenotypes [94, 95]. Therefore, these cumulative observations have validated STAT3 as a novel target for cancer therapy, and hence provided the rationale for developing small-molecule STAT3 inhibitors. We have recently reported that alantolactone inhibits STAT3 activation in HepG2 cells [50], but more investigations are required to fully address the role of alantolactone and isoalantolactone on STAT3 and its regulated gene products in cancer cells.

## 5. Concluding Remarks and Future Perspectives

In this review, we summarized the recent progress of both alantolactone and isoalantolactone with cytotoxic activities. Many studies have shown that alantolactone and isoalantolactone induce apoptosis of many types of cancer cells. However, reports on underlying mechanism of actions of these compounds are limited. According to the literature and also to our previous results, data compiled in Table 1 summarize the major molecular targets that have been characterized thus far. *In vivo* toxicological studies on alantolactone and isoalantolactone demonstrated that these compounds did not induce significant hepatotoxicity and nephrotoxicity in mice, and their ability to cross the blood brain barrier. References [49, 50, 52] provides evidence that these are potential lead compounds for future anticancer drug development or may serve as chemical templates for the design, synthesis, and semisynthesis of new substances for the treatment of cancer. Further investigations are needed on how they specifically induce apoptosis in cancers and spare normal cells. Additional studies are yet required to elucidate the full spectrum of cytotoxic activities of these compounds to validate in preclinical and clinical applications and to make clear the potential role of alantolactone and isoalantolactone as potent anticancer agents.

## Figures and Tables

**Table 1:** Molecular targets of alantolactone in different cancer types.

Cancer type	Cell lines	EC50/concentration	Molecular targets	References
Liver	HepG2, Bel-7402, SMMC-7721	40 *µ*M	Bax/Bcl-2↑, caspase-3↑, STAT3⊥, caspase-8↑, MMP↓, Bid↑, NF-*κ*B/p65↓	[50, 51]
Glioblastoma	U87	20 and 40 *µ*M	p53↑, Bax↑, Bcl-2↓, caspase-9↑, caspase-3↑, ADP-ribose↓, NF-*κ*B⊥, ROS↑	[49]
Colon	HCT-8	5 *µ*g/mL	activin/SMAD3 signaling↑, Cripto-1/ActRII⊥	[45]
Leukemia	HL-60, K562, K562/ADR	10, 25, and 50 *µ*g/mL	ROS↑, cytochrome-c↑, Bax↑, PARP↓, ADP-ribose↓, NF-*κ*B⊥, DNA-binding↓, I*κ*B*α* phosphorylation↑, p21↑, Bcr/Abl↓, P-glycoprotein↓, cyclin B1↓, cyclin-dependent protein kinase-1↓,	[32, 56, 57]
Lung	A549	6.25, 12.5, and 25 *µ*M/L	—	[53]
Other cancers	MK-1, HeLa, and B16F10	—	—	[15]

↓: Downregulation; ↑: Upregulation; ⊥: Inhibition.

## References

[1] Koehn FE, Carter GT (2005). The evolving role of natural products in drug discovery. *Nature Reviews Drug Discovery*.

[2] Cragg GM, Newman DJ (2005). Plants as a source of anti-cancer agents. *Journal of Ethnopharmacology*.

[3] Christen P, Cuendet M (2012). Plants as a source of therapeutic and health products. *Chimia*.

[4] Koehn FE, Carter GT (2005). Rediscovering natural products as a source of new drugs. *Discovery Medicine*.

[5] Amin ARMR, Kucuk O, Khuri FR, Shin DM (2009). Perspectives for cancer prevention with natural compounds. *Journal of Clinical Oncology*.

[6] Pezzuto JM (1997). Plant-derived anticancer agents. *Biochemical Pharmacology*.

[7] Kreuger MR, Grootjans S, Biavatti MW, Vandenabeele P, D'Herde K (2012). Sesquiterpene lactones as drugs with multiple targets in cancer treatment: focus on parthenolide. *Anticancer Drugs*.

[8] Heinrich M, Robles M, West JE, Ortiz de Montellano BR, Rodriguez E (1998). Ethnopharmacology of Mexican asteraceae (compositae). *Annual Review of Pharmacology and Toxicology*.

[9] Rasul A, Perveen S, Ma T (2012). Costunolide: a novel anti-cancer sesquiterpene lactone. *Bangladesh Journal of Pharmacology*.

[10] Zhang S, Won Y-K, Ong C-N, Shen H-M (2005). Anti-cancer potential of sesquiterpene lactones: bioactivity and molecular mechanisms. *Current Medicinal Chemistry*.

[11] Gu J-Q, Gills JJ, Park EJ (2002). Sesquiterpenoids from *Tithonia diversifolia* with potential cancer chemopreventive activity. *Journal of Natural Products*.

[12] Koch E, Klaas CA, Rungeler P (2001). Inhibition of inflammatory cytokine production and lymphocyte proliferation by structurally different sesquiterpene lactones correlates with their effect on activation of NF-*κ*B. *Biochemical Pharmacology*.

[13] Nam NH (2006). Naturally occurring NF-*κ*B inhibitors. *Mini Reviews in Medicinal Chemistry*.

[14] Lim SS, Kim JR, Lim HA (2007). Induction of detoxifying enzyme by sesquiterpenes present in *Inula helenium*. *Journal of Medicinal Food*.

[15] Konishi T, Shimada Y, Nagao T, Okabe H, Konoshima T (2002). Antiproliferative sesquiterpene lactones from the roots of *Inula helenium*. *Biological and Pharmaceutical Bulletin*.

[16] Blagojevic PD, Radulovic NS (2012). Conformational analysis of antistaphylococcal sesquiterpene lactones from *Inula helenium* essential oil. *Natural Product Communications*.

[17] Huo Y, Shi H, Li W, Wang M, Li X (2010). HPLC determination and NMR structural elucidation of sesquiterpene lactones in *Inula helenium*. *Journal of Pharmaceutical and Biomedical Analysis*.

[18] Ketai W, Huitao L, Yunkun Z (2000). Separation and determination of alantolactone and isoalantolactone in traditional Chinese herbs by capillary electrophoresis. *Talanta*.

[19] Seo JY, Lim SS, Kim JR (2008). Nrf2-mediated induction of detoxifying enzymes by alantolactone present in *Inula helenium*. *Phytotherapy Research*.

[20] Seo JY, Park J, Kim HJ (2009). Isoalantolactone from *Inula helenium* caused nrf2-mediated induction of detoxifying enzymes. *Journal of Medicinal Food*.

[21] Stojakowska A, Michalska K, Malarz J (2006). Simultaneous quantification of eudesmanolides and thymol derivatives from tissues of *Inula helenium* and I. royleana by reversed-phase high-performance liquid chromatography. *Phytochemical Analysis*.

[22] Stojanovic-Radic Z, Comic L, Radulovic N (2012). Antistaphylococcal activity of *Inula helenium* L. root essential oil: eudesmane sesquiterpene lactones induce cell membrane damage. *European Journal of Clinical Microbiology & Infectious Diseases*.

[23] Li Y, Ni ZY, Zhu MC (2012). Antitumour activities of sesquiterpene lactones from *Inula helenium* and *Inula japonica*. *Zeitschrift für Naturforschung C*.

[24] Chun J, Choi RJ, Khan S (2012). Alantolactone suppresses inducible nitric oxide synthase and cyclooxygenase-2 expression by down-regulating NF-*κ*B, MAPK and AP-1 via the MyD88 signaling pathway in LPS-activated RAW 264.7 cells. *International Immunopharmacology*.

[25] Cantrell CL, Abate L, Fronczek FR, Franzblau SG, Quijano L, Fischer NH (1999). Antimycobacterial eudesmanolides from *Inula helenium* and *Rudbeckia subtomentosa*. *Planta Medica*.

[26] Wenhua G, Yaowen C, Yegao Y, Xingguo C, Zhide H (2004). Separation and determination of two sesquiterpene lactones in *Radix inulae* and Liuwei Anxian San by microemulsion electrokinetic chromatography. *Biomedical Chromatography*.

[27] Cantrell CL, Pridgeon JW, Fronczek FR, Becnel JJ (2010). Structure-activity relationship studies on derivatives of eudesmanolides from *Inula helenium* as toxicants against *Aedes aegypti* larvae and adults. *Chemistry and Biodiversity*.

[28] Tan RX, Tang HQ, Hu J, Shuai B (1998). Lignans and sesquiterpene lactones from *Artemisia sieversiana* and *Inula racemosa*. *Phytochemistry*.

[29] Tripathi YB, Tripathi P, Upadhyay BN (1988). Assessment of the adrenegric beta-blocking activity of *Inula racemosa*. *Journal of Ethnopharmacology*.

[30] Xin X-L, Ma X-C, Liu K-X, Han J, Wang B-R, Guo D-A (2008). Microbial transformation of alantolactone by Mucor polymorphosporus. *Journal of Asian Natural Products Research*.

[31] Schmidt TJ, Brun R, Willuhn G, Khalid SA (2002). Anti-trypanosomal activity of helenalin and some structurally related sesquiterpene lactones. *Planta Medica*.

[32] Pal HC, Sehar I, Bhushan S, Gupta BD, Saxena AK (2010). Activation of caspases and poly (ADP-ribose) polymerase cleavage to induce apoptosis in leukemia HL-60 cells by *Inula racemosa*. *Toxicology in Vitro*.

[33] Qiu J, Luo M, Wang J (2011). Isoalantolactone protects against *Staphylococcus aureus* pneumonia. *FEMS Microbiology Letters*.

[34] Elmore S (2007). Apoptosis: a review of programmed cell death. *Toxicologic Pathology*.

[35] Hengartner MO (2000). The biochemistry of apoptosis. *Nature*.

[36] Evan GI, Vousden KH (2001). Proliferation, cell cycle and apoptosis in cancer. *Nature*.

[37] Hanahan D, Weinberg RA (2000). The hallmarks of cancer. *Cell*.

[38] Rasul A, Bao R, Malhi M (2013). Induction of apoptosis by costunolide in bladder cancer cells is mediated through ROS generation and mitochondrial dysfunction. *Molecules*.

[39] Rasul A, Ding C, Li X (2012). Dracorhodin perchlorate inhibits PI3K/Akt and NF-*κ*B activation, up-regulates the expression of p53, and enhances apoptosis. *Apoptosis*.

[40] Rasul A, Khan M, Yu B, Ma T, Yang H (2011). Xanthoxyletin, a coumarin induces S phase arrest and apoptosis in human gastric adenocarcinoma SGC-7901 cells. *Asian Pacific Journal of Cancer Prevention*.

[41] Rasul A, Song R, Wei W (2012). Tubeimoside-1 inhibits growth via the induction of cell cycle arrest and apoptosis in human melanoma A375 cells. *Bangladesh Journal of Pharmacology*.

[42] Rasul A, Yu B, Khan M (2012). Magnolol, a natural compound, induces apoptosis of SGC-7901 human gastric adenocarcinoma cells via the mitochondrial and PI3K/Akt signaling pathways. *International Journal of Oncology*.

[43] Rasul A, Yu B, Yang L-F (2011). Induction of mitochondria-mediated apoptosis in human gastric adenocarcinoma SGC-7901 cells by kuraridin and Nor-kurarinone isolated from *Sophora flavescens*. *Asian Pacific Journal of Cancer Prevention*.

[44] Rasul A, Yu B, Zhong L, Khan M, Yang H, Ma T (2012). Cytotoxic effect of evodiamine in SGC-7901 human gastric adenocarcinoma cells via simultaneous induction of apoptosis and autophagy. *Oncology Reports*.

[45] Shi Y, Bao YL, Wu Y (2011). Alantolactone inhibits cell proliferation by interrupting the interaction between Cripto-1 and activin receptor type II A in activin signaling pathway. *Journal of Biomolecular Screening*.

[46] Fulda S, Debatin K-M (2006). Extrinsic versus intrinsic apoptosis pathways in anticancer chemotherapy. *Oncogene*.

[47] Manly SP, Padmanabha R, Lowe SE (2002). Natural products or not? How to screen for natural products in the emerging HTS paradigm. *Methods in Molecular Biology*.

[48] Lawrence NJ, McGown AT, Nduka J, Hadfield JA, Pritchard RG (2001). Cytotoxic Michael-type amine adducts of *α*-methylene lactones alantolactone and isoalantolactone. *Bioorganic and Medicinal Chemistry Letters*.

[49] Khan M, Yi F, Rasul A (2012). Alantolactone induces apoptosis in glioblastoma cells via GSH depletion, ROS generation, and mitochondrial dysfunction. *IUBMB Life*.

[50] Khan M, Li T, Ahmad Khan MK (2013). Alantolactone induces apoptosis in HepG2 cells through GSH depletion, inhibition of STAT3 activation, and mitochondrial dysfunction. *BioMed Research International *.

[51] Lei JC, Yu JQ, Yin Y, Liu YW, Zou GL (2012). Alantolactone induces activation of apoptosis in human hepatoma cells. *Food and Chemical Toxicology*.

[52] Khan M, Ding C, Rasul A (2012). Isoalantolactone induces reactive oxygen species mediated apoptosis in pancreatic carcinoma PANC-1 cells. *International Journal of Biological Sciences*.

[53] Min-ru Z, Ying-hao Z, Kun Z, Long-fei Y, Yong-chen Z, Cheng-yan H (2011). Inhibitory effects of natural compound alantolactone on human non-small cell lung cancer A549 cells. *Chemical Research in Chinese Universities*.

[54] Buytaert E, Dewaele M, Agostinis P (2007). Molecular effectors of multiple cell death pathways initiated by photodynamic therapy. *Biochimica et Biophysica Acta*.

[55] Birt DF, Hendrich S, Wang W (2001). Dietary agents in cancer prevention: flavonoids and isoflavonoids. *Pharmacology and Therapeutics*.

[56] Wei W, Huang H, Zhao S (2013). Alantolactone induces apoptosis in chronic myelogenous leukemia sensitive or resistant to imatinib through NF-*κ*B inhibition and Bcr/Abl protein deletion. *Apoptosis*.

[57] Yang C, Yang J, Sun M, Yan J, Meng X, Ma T (2013). Alantolactone inhibits growth of K562/adriamycin cells by downregulating Bcr/Abl and P-glycoprotein expression. *IUBMB Life*.

[58] Slater AF, Stefan C, Nobel I, van den Dobbelsteen DJ, Orrenius S (1995). Signalling mechanisms and oxidative stress in apoptosis. *Toxicology Letters*.

[59] Jae HL, Baek N-I, Kim S-H (2007). A new cytotoxic prenylated chalcone from Sophora flavescens. *Archives of Pharmacal Research*.

[60] Vercesi AE, Kowaltowski AJ, Grijalba MT, Meinicke AR, Castilho RF (1997). The role of reactive oxygen species in mitochondrial permeability transition. *Bioscience Reports*.

[61] Cohen GM (1997). Caspases: the executioners of apoptosis. *Biochemical Journal*.

[62] Adams JM (2003). Ways of dying: multiple pathways to apoptosis. *Genes and Development*.

[63] Porter AG, Janicke RU (1999). Emerging roles of caspase-3 in apoptosis. *Cell Death and Differentiation*.

[64] Kluck RM, Bossy-Wetzel E, Green DR, Newmeyer DD (1997). The release of cytochrome C from mitochondria: a primary site for Bcl-2 regulation of apoptosis. *Science*.

[65] Fulda S (2010). Evasion of apoptosis as a cellular stress response in cancer. *International Journal of Cell Biology*.

[66] Lawen A (2003). Apoptosis—an introduction. *BioEssays*.

[67] Reed JC (2002). Apoptosis-based therapies. *Nature Reviews Drug Discovery*.

[68] Budram-Mahadeo V, Morris PJ, Latchman DS (2002). The Brn-3a transcription factor inhibits the pro-apoptotic effect of p53 and enhances cell cycle arrest by differentially regulating the activity of the p53 target genes encoding Bax and p21^CIP1/Waf1^. *Oncogene*.

[69] Fridman JS, Lowe SW (2003). Control of apoptosis by p53. *Oncogene*.

[70] Vogelstein B, Lane D, Levine AJ (2000). Surfing the p53 network. *Nature*.

[71] Vogelstein B, Kinzler KW (2001). Achilles’ heel of cancer?. *Nature*.

[72] Lu C, El-Deiry WS (2009). Targeting p53 for enhanced radio- and chemo-sensitivity. *Apoptosis*.

[73] Ghosh S, Hayden MS (2008). New regulators of NF-*κ*B in inflammation. *Nature Reviews Immunology*.

[74] Karin M, Cao Y, Greten FR, Li Z-W (2002). NF-*κ*B in cancer: from innocent bystander to major culprit. *Nature Reviews Cancer*.

[75] Lin W-W, Karin M (2007). A cytokine-mediated link between innate immunity, inflammation, and cancer. *Journal of Clinical Investigation*.

[76] Luo J-L, Kamata H, Karin M (2005). IKK/NF-*κ*B signaling: balancing life and death—a new approach to cancer therapy. *Journal of Clinical Investigation*.

[77] Affara NI, Coussens LM (2007). IKK*α* at the crossroads of inflammation and metastasis. *Cell*.

[78] Karin M (2008). The I*κ*B kinase—a bridge between inflammation and cancer. *Cell Research*.

[79] Levidou G, Korkolopoulou P, Nikiteas N (2007). Expression of nuclear factor *κ*B in human gastric carcinoma: relationship with I*κ*B a and prognostic significance. *Virchows Archiv*.

[80] Shen H-M, Tergaonkar V (2009). NF*κ*B signaling in carcinogenesis and as a potential molecular target for cancer therapy. *Apoptosis*.

[81] Bharti AC, Donato N, Singh S, Aggarwal BB (2003). Curcumin (diferuloylmethane) down-regulates the constitutive activation of nuclear factor-*κ*B and I*κ*B*α* kinase in human multiple myeloma cells, leading to suppression of proliferation and induction of apoptosis. *Blood*.

[82] Li Q, Yu Y-Y, Zhu Z-G (2005). Effect of NF-*κ*B constitutive activation on proliferation and apoptosis of gastric cancer cell lines. *European Surgical Research*.

[83] Klippel A, Reinhard C, Kavanaugh WM, Apell G, Escobedo M-A, Williams LT (1996). Membrane localization of phosphatidylinositol 3-kinase is sufficient to activate multiple signal-transducing kinase pathways. *Molecular and Cellular Biology*.

[84] Kauffmann-Zeh A, Rodriguez-Viciana P, Ulrich E (1997). Suppression of c-Myc-induced apoptosis by Ras signalling through PI(3)K and PKB. *Nature*.

[85] Han Z, Hong L, Han Y (2007). Phospho Akt mediates multidrug resistance of gastric cancer cells through regulation of P-gp, Bcl-2 and Bax. *Journal of Experimental and Clinical Cancer Research*.

[86] Jeong W-S, Jun M, Kong A-NT (2006). Nrf2: a potential molecular target for cancer chemoprevention by natural compounds. *Antioxidants and Redox Signaling*.

[87] Sano S, Itami S, Takeda K (1999). Keratinocyte-specific ablation of Stat3 exhibits impaired skin remodeling, but does not affect skin morphogenesis. *The EMBO Journal*.

[88] Silver DL, Montell DJ (2001). Paracrine signaling through the JAK/STAT pathway activates invasive behavior of ovarian epithelial cells in drosophila. *Cell*.

[89] Ma X-T, Wang S, Ye Y-J, Du R-Y, Cui Z-R, Somsouk M (2004). Constitutive activation of Stat3 signaling pathway in human colorectal carcinoma. *World Journal of Gastroenterology*.

[90] Mora LB, Buettner R, Seigne J (2002). Constitutive activation of Stat3 in human prostate tumors and cell lines: direct inhibition of Stat3 signaling induces apoptosis of prostate cancer cells. *Cancer Research*.

[91] Turkson J, Jove R (2000). STAT proteins: novel molecular targets for cancer drug discovery. *Oncogene*.

[92] Udayakumar TS, Nagle RB, Bowden GT (2004). Fibroblast growth factor-I transcriptionally induces membrane type-I matrix metalloproteinase expression in prostate carcinoma cell line. *Prostate*.

[93] Xie T-X, Wei D, Liu M (2004). Stat3 activation regulates the expression of matrix metalloproteinase-2 and tumor invasion and metastasis. *Oncogene*.

[94] Nagakawa O, Murakami K, Yamaura T (2000). Expression of membrane-type 1 matrix metalloproteinase (MT1-MMP) on prostate cancer cell lines. *Cancer Letters*.

[95] Udayakumar TS, Chen ML, Bair EL (2003). Membrane type-1-matrix metalloproteinase expressed by prostate carcinoma cells cleaves human laminin-5 *β*3 chain and induces cell migration. *Cancer Research*.

